# Euthanasia in Africa: A scoping review of empirical evidence

**DOI:** 10.1002/hsr2.1239

**Published:** 2023-05-30

**Authors:** Jimoh Amzat, Kehinde Kazeem Kanmodi, Abbas Ismail, Eyinade Adeduntan Egbedina

**Affiliations:** ^1^ Department of Sociology Usmanu Danfodiyo University Sokoto Nigeria; ^2^ Department of Sociology University of Johannesburg Johannesburg South Africa; ^3^ School of Health and Life Sciences Teesside University Middlesbrough UK; ^4^ Faculty of Dentistry University of Puthisastra Phnom Penh Cambodia; ^5^ Cephas Health Research Initiative Inc Ibadan Nigeria; ^6^ Department of Sociology Umaru Musa Yar'adua University Katsina Nigeria

**Keywords:** Africa, euthanasia, mercy killing, scoping review

## Abstract

**Background and Aims:**

The core ethical perplexity is that physician‐assisted suicide and euthanasia (PAS/E) contradicts the core value of medical practice which is about the duty of care to preserve life. While most arguments for and against euthanasia emerge from other continents, no African country legalizes or decriminalizes PAS/E. The essence of this scoping review is to collate evidence and scientific voices on euthanasia in Africa by synthesizing empirical articles on the subject in Africa.

**Methods:**

In this scoping review, a systematic search of five electronic research databases—PubMed, SCOPUS, CINHAL Complete, Allied and Complementary Medicine (AMED), and APA PsycInfo—was conducted to identify relevant articles conducted in Africa on euthanasia. After deduplication with the Rayyan software, the retrieved literature were screened for eligibility, and only eligible articles were included in the review. Relevant data from these articles were extracted and analyzed using narrative synthesis.

**Results:**

Only 14 articles reporting empirical studies, conducted in Africa, and published in English, were included in the review after a rigorous screening process. The review shows a wide rejection of euthanasia, but there is not much resistance to passive euthanasia, that is, withholding/withdrawing life‐saving medical care from a terminally ill patient, mostly due to advanced age of the patient and the incurability of the illness. Many factors, such as religion, profession, and age help in shaping the way an individual view and understand PAS/E. Professionals take the patient's clinical condition and sociocultural context into consideration when making decisions about end‐of‐life care. The sociocultural context did not favor PAS/E.

**Conclusion:**

Euthanasia will continue to be a subject of controversy and debate in Africa and elsewhere. The majority of Africans hold the duty of care and preservation of life as the hallmark of medical practice, which informs the wide rejection of PAS/E.

## 1 INTRODUCTION

Euthanasia literarily means “good death” and it is a conscious and deliberate termination of a patient's life by a physician due to a terminal or incurable illness.^1^ Euthanasia is a bit different from physician‐assisted suicide (PAS) because, in the latter, the physician provides the means which is self‐administered by the patient. The result and purpose are the same—the termination of a terminally‐ill patient's life. There is another different but close concept, which is physician‐assisted suicide, which might not be in a medical context or for a terminally ill person. While all of them are forms of physician‐assisted dying or medically assisted death, the focus of this review is on physician‐assisted suicide and euthanasia (PAS/E), which are forms of active euthanasia that is, deliberate provision of means or administration of a lethal drug. On the other hand, there is passive euthanasia, which includes withholding and withdrawal of treatment following a poor prognosis or chance of survival.^1^, ^2^, ^3^ There is strong ethical debate against PAS/E; hence, it is not a welcome practice in most countries except in Belgium, the Netherlands, Luxembourg, and Switzerland (among others) where it has been decriminalized that is, permissible (after due medical diligence) without a legal right to it.

The core ethical perplexity is that the PAS/E contradicts the core value of medical practice which is about the duty of care to preserve life.^4^, ^5^ The care setting should be a space of hope for the ill, not a place where the supposed caregiver will actively participate or assist in dying. The essence of this study is not to go into the unending (ethical) debate about PAS/E. The ethical arguments are in abundance, although mostly against,^6^, ^7^, ^8^ some favor PAS/E.^9^ While most arguments for and against euthanasia emerge from other continents, there is no country in Africa which legalizes or decriminalizes PAS/E. The legal framework is not enough to dispel the debate or voices about PAS/E in Africa. The essence of this review is to collate evidence and scientific voices on euthanasia in Africa by summarizing empirical articles on the subject in Africa.

## 2 METHODS

### 2.1 Review design

The methodology of this study adopted the design recommended by Arksey and O'Malley^10^ which proposed the following steps for conducting a scoping review: research question identification (Step 1); relevant literature identification (step 2); literature selection (step 3); data charting (step 4); and collation, summarization, and presentation of results (step 5).^10^

### 2.2 Identification of research question

The research question of this scoping review was: What does the existing literature reveal concerning euthanasia in Africa?

### 2.3 Identification of relevant literature

The search strategy adopted in this scoping review was based on the PCC (Population [P], Concept [C], and Context [C]) framework.^11^ The population of interest was African population, the concept was euthanasia, and the context was African countries, territories, and dependencies.

On 12 January 2023, a systematic search of five electronic research databases—PubMed, SCOPUS, CINHAL Complete, Allied and Complementary Medicine (AMED), and APA PsycInfo—was done, with the aid of Boolean operators, using the names of African countries, territories and dependencies, and euthanasia and its synonyms (“assisted suicide” and “mercy killing”) (Tables A1, A2, A3 [Appendix]).

### 2.4 Selection of literature

The literature retrieved from the database search was imported to Rayyan software for deduplication. After deduplication, all the remaining literature was subjected to a two‐stage screening process, done by two independent reviewers, to identify relevant literature eligible for inclusion in the scoping review. Below are the criteria used for the inclusion or exclusion of the screened literature:

#### 2.4.1 Inclusion criteria

- Literature published in peer‐reviewed journals.
- Literature reporting empirical studies of any research design.
- Literature published in English language.
- Literature investigating the concept of euthanasia in Africa.

#### 2.4.2 Exclusion criteria

- Literature published in non‐peer‐reviewed journals.
- Peer‐reviewed literature not reporting empirical studies.
- Literature published in non‐English language.
- Literature not investigating the concept of euthanasia in Africa.
- Literature investigating the concept of euthanasia in a study location outside the African continent.
- Literature with inaccessible full text.

The first stage screening process involved title and abstract screening—in this stage, clearly nonrelevant articles were excluded while the remaining (non‐excluded) literature were considered for the second stage screening. The second stage screening involved the evaluation of the content of the full texts. Only the articles that met the review's inclusion criteria were included in the scoping review.

### 2.5 Charting of data

Data were extracted from the included literature for analysis; these data included citation details (names of authors and the year of publication), study type, aims of the study, study population size and characteristics, location of the study, population of the study, and relevant findings that address the scoping review question.

### 2.6 Collation, summarization, and presentation of results

The extracted data were collated, summarized, and presented using texts and a table.

### 2.7 Ethical considerations

Being a scoping review, ethical approval is not applicable to this study, as this study did not collect data from human or animal subjects but from an open research repository.

## 3 RESULTS

**Figure 1:**
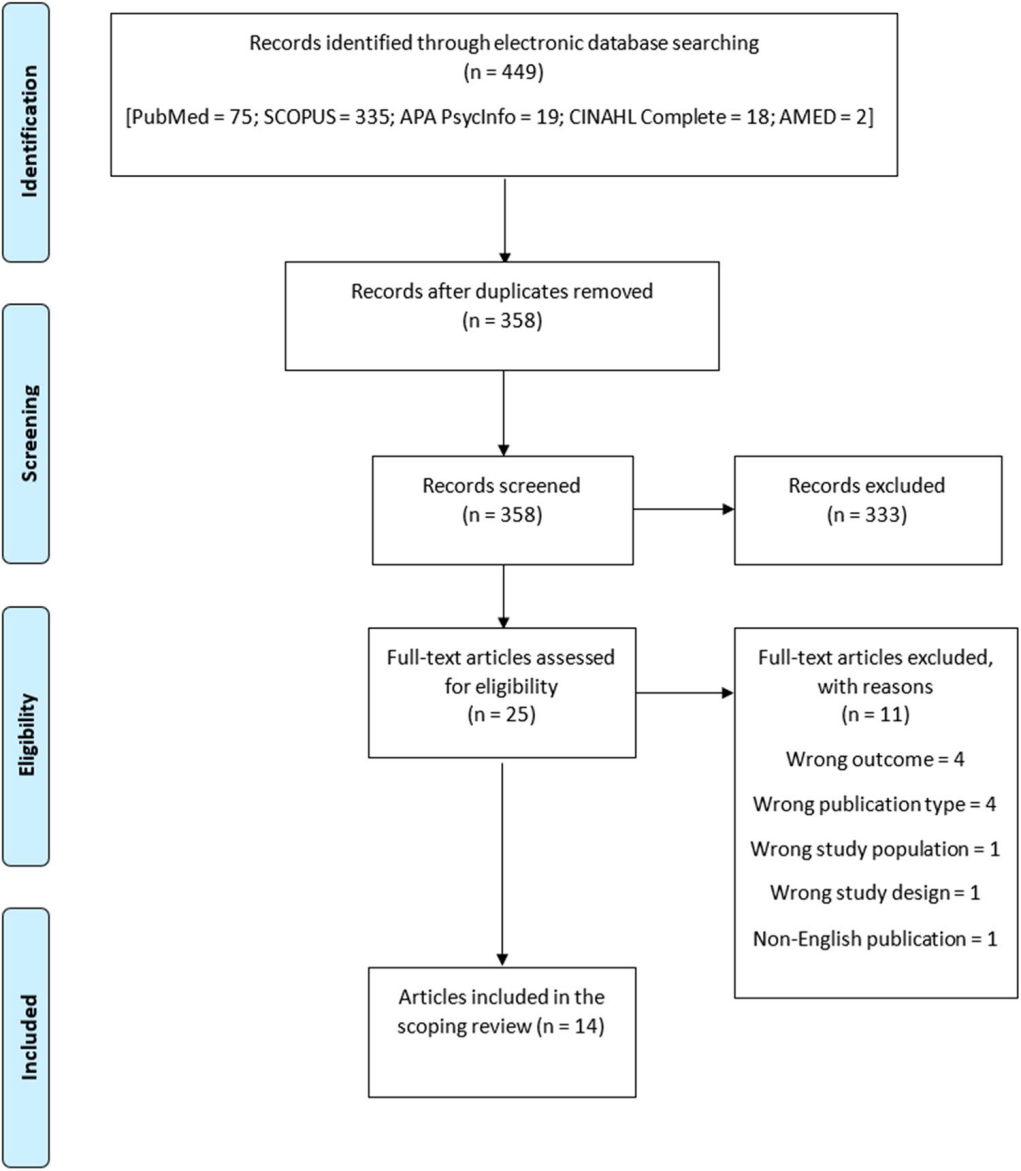
Flow chart of literature search and sorting process.

A total of 499 literature items were retrieved from the database search, of which 91 were duplicate literature and removed. From the remaining 358 literature, 333 were excluded after the title and abstract screening. After the full‐text screening of the remaining 25 literature (Table A4 [Appendix]), only 14 articles were found eligible for inclusion in the scoping review (Figure 1).

### 3.1 Studied populations

Each of the reviewed papers focused on different population groups: doctors, nurses, and mothers,^12^ medical students,^13^, ^14^ junior and senior doctors,^15^ and senior clinicians.^16^ Other sets of population include private medical practitioners,^17^ physicians,^18^ psychiatrists,^19^ aged adults^20^ and adults.^21^ Some papers include other population groups containing the Xhosa ethnic group of South Africa,^22^ lay people and health professionals,^23^ all patients had life support,^24^ oncologists, and registered nurses.^25^ The majority of the study population is made up of physicians and other healthcare workers from different specialties.

### 3.2 Study design

The reviewed papers adopted different methodologies in trying to achieve their aims. Some of them adopted descriptive cross‐sectional design.^12^, ^14^, ^17^, ^22^ Others adopted different designs that included observational study,^18^ prospective survey^24^ and other designs as presented in Table 1. Seven of the studies were carried out in a hospital setting.^12^, ^15^, ^16^, ^18^, ^24^, ^25^

**Table 1:** Summary of selected publications on euthanasia/pas in africa.

S/No.	Author and date	Sample size	Location/country	Objectives	Study design	Population	Conclusion
1.	[12]	70 mothers, 33 doctors, and 20 nurses	Baragwanath hospital, Soweto, 15 km from Johannesburg, South Africa	To gain insight into the attitudes of doctors, nurses and mothers of recent neonatal intensive care unit (NICU) survivors toward the utilization and withdrawal of life support in the context of a developing society	Descriptive cohort study	Doctors, nurses and mothers	Resource allocation needs to be an integral component of NICU in developing nations due to the nonideal conditions in which the doctors and nurses in those settings work.
2.	[13]	270	Faculty of Medicine, University of Khartoum, Khartoum, Sudan	To investigate the attitudes of the final year medical students of a Sudanese university toward euthanasia, and to determine factors that influence these attitudes	Cross‐sectional descriptive survey	Final‐year students of the Faculty of Medicine, University of Khartoum, Khartoum, Sudan.	There was no formal teaching about euthanasia in the medical school. There is urgent need to update or change the curriculum to cover euthanasia and PAS in medical schools
3.	[15]	248	Khartoum Teaching Hospital and Omdurman Teaching Hospital, Sudan	To determine the attitude of junior and senior Sudanese doctors to euthanasia and assisted suicide	Cross‐sectional descriptive survey	Junior and senior doctors	Doctors within the country are conservative and reject euthanasia and PAS
4.	[16]	67	University of Benin Teaching Hospital (UBTH), Nigeria	To examine the attitude of clinicians involved in care to the terminally ill patients in our hospital and community generally	Descriptive study	Senior clinicians (11 females and 56 males)	There is opposition to PAS to the terminally ill patients and majority of them will not support life during palliative care. There is disjuncture between what clinicians think and what is practiced
5.	[17]	100	Bloemfontein, South Africa	To determine the opinions of private medical practitioners in Bloemfontein, South Africa, regarding euthanasia of terminally ill patients	Descriptive study	Private medical practitioners in Bloemfontein.	There was strong opposition to prescribing of medication to let the patient die, and withdrawal of essential medical treatment to speed up death was the most acceptable method. Patient, his doctor and close family members should be included in the decision making processes of choosing the best option
6.	[18]	306	At the major hospitals in the southern, central, and northern regions of the country— Maputo, Beira, and Nampula—as well as Xai‐Xai Provincial Hospital (XXPH), Mozambique	To evaluate the general knowledge, attitudes, and practices of Mozambican physicians on palliative care.	Cross–sectional observational study	Physicians	The doctors at Mozambique's three primary hospitals and the only provincial hospital with a separate palliative care service have a basic understanding of palliative care. The family‐centered model and paternalism are most common. Medical ethical principles are used to guide decision‐making in each unique situation, particularly when the patient is no longer competent.
7.	[19]	160 psychiatrists	4 selected counties in Egypt—Cairo, Giza, Helwan, Alexandria	To survey the views of Egyptian psychiatrist on physician‐assisted suicide, focusing on demographical, spiritual, legal and clinical domains.	Descriptive study	Psychiatrists	The majority of respondents (80%) were against PAS. the fact that there are no appreciable distinctions between Muslims and Christians in Egypt in terms of psychiatrists' opinions. This may suggest that the views of Egyptian psychiatrists are more strongly influenced by culture than by religion.
8.	[20]	120	It was carried out among the older adults that resides in the 8 homes for the aged in rural and urban areas of Cape Town, South Africa	To establish the influence of cultural background, purpose in life, health and age on the attitudes of older adults towards euthanasia.	Descriptive study	Older adults	Decisions about the end of life are deeply personal and unpredictable. Therefore, healthcare professionals should be very sensitive to each person's motivations and justifications for making an end‐of‐life decision.
9.	[21]	80	Buganda, a subnational kingdom within Uganda	To explore attitudes towards euthanasia among Ugandan adults.	Descriptive study	Adults	Almost all participants (97 percent) perceived euthanasia as murder. Overwhelming majority of the participants opposed euthanasia. Religion and sociocultural factors influence the stand of the participants. It is perceived as morally wrong and the general perception in this study was that pain should not justify the right to die. Legalizing euthanasia is perceived as a license to kill. Findings also show that participants opposed euthanasia due to hope for new medical discoveries, elevated by religious beliefs.
10.	[22]	163	Transkei, South Africa	The attitudes toward death and dying of 163 Xhosa‐speaking people living in Transkei, South Africa	Exploratory descriptive study	The Xhosa ethnic group of South Africa	The majority of respondents, irrespective of whether they believed in an afterlife or not, expressed their fear of death. There is lack of consensus concerning euthanasia. Many considered it an immoral act.
11.	[23]	312 lay people and 198 health professionals	Lomé, the capital city of Togo, Togo	To study the views on the acceptability of Physician‐assisted‐suicide (PAS) of lay people and health Professionals in an African country, Togo.		Lay people and health professionals	Most Togolese laypeople are neither categorically in favor of PAS nor opposed to it; rather, they assess its level of acceptability in light of specific circumstances.
12.	[24]		ICUs in tertiary centers in London and Cape Town.	To examine the frequency of limiting (withdrawing and withholding) therapy in the intensive care unit (ICU), the grounds for limiting therapy, the people involved in the decisions, the way the decisions are implemented and the patient outcome.	Prospective survey	All patients who died or had life support limited	Withdrawal from therapy happened frequently, most frequently due to failure of multiple organs. Before a decision was made, there was broad agreement, and deaths generally occurred quickly.
13.	[25]	6	Two private hospitals in Port Elizabeth	To explore and describe the experience of Registered Nurses regarding the withdrawal of treatment from the critically ill patient in an Intensive Care Unit (ICU)	Qualitative, descriptive, exploratory	Registered Nurses	Many nurses experiencing the situation of treatment withdrawal appear to experience it in isolation. Many of the nurses employ such good defense mechanisms that they are reluctant to actually share how they are really feeling.
14.	[14]	277	Stellenbosch University, South Africa	To determine the views of future doctors (medical students) regarding euthanasia and physician‐assisted suicide (PAS) and to ascertain their stance on its legalization in South Africa.	Semi‐quantitative descriptive study design	Third‐ to fifth‐year medical students	Comparatively more respondents to this study than to earlier ones were in favor of legalizing PAS or euthanasia. Only 41.9% of respondents, however, said they would actually consider performing PAS or euthanasia on some patients. Before policy can be informed, viewpoints from the public and other healthcare professionals are required.

### 3.3 Study setting

Others were conducted in a school environment^13^, ^14^ There is another study conducted in homes for the elderly in Egypt,^20^ hospital setting^12^, ^15^, ^16^, ^18^, ^24^, ^25^ and in other settings that the study found suitable (see Table 1).

### 3.4 Factors influencing attitudes toward euthanasia

A variety of factors, including personal philosophy, sex, religious and cultural teachings, and prior contact with terminally‐ill patients, determine the attitudes of both medical and nonmedical research participants toward PAS/E in Africa. The central cultural and religious context is about the sanctity of life. “While there is life, there is hope” is a widely held belief that significantly affects how people view euthanasia in Africa. Life is revered as sacred and should only be taken by God. In Sudan, several doctors held the opinion that PAS/E was never morally acceptable, but few others thought it might be in certain circumstances.^12^, ^15^

Most medical professionals oppose PAS/E, mostly due to religious reasons which have a greater impact on their medical practice. The implication is that nonclinical factors play a major role in forming the attitude toward PAS/E. The practice is perceived as a sin. There is always “hope for recovery” with divine intervention even when the condition is irreversible. As such, there is no reason for ending one's life. Instead, clinicians were much more open to delaying or stopping treatment than to PAS/E.^14^, ^19^, ^21^

In spite of the general rejection of PAS/E in Africa, the review showed less resistance to withholding life‐saving medical care from terminally ill patients^14^, ^17^—conceptually described as passive euthanasia. There are minority reports of a positive attitude toward euthanasia which was significantly correlated with a terminal illness in old age.^20^, ^22^ It cannot be assumed that as one ages, one will continue to adhere to religious doctrines and worldviews about PAS/E. The individual patient may conceal the suffering brought on by an age‐related illness, yet consider euthanasia as a means of achieving a final peace.^20^, ^22^

The advanced age of the patient and the incurability of the illness were the most significant factors in the positive attitude toward euthanasia.^20^, ^22^ It is anticipated that those who have endured excruciating pain and a long‐term medical condition might consent to PAS/E to find a permanent cure for their persistent condition.^23^ On the other hand, healthy young people wanted to be informed if they were in a situation where they suspected they had a terminal illness so that a decision and the appropriate course of action could be taken.^22^

### 3.5 Knowledge and information sharing on euthanasia and decisions on end of life

The review shows that only a few doctors and medical students are knowledgeable about all euthanasia‐related concepts.^13^, ^18^ In spite of this low knowledge, the health workers were in a position to communicate with the patients about their diagnosis, prognosis and available medical options. The review noted that some medical professionals would rather inform the patient's family members of the prognosis. This will allow the patient's family to comfort him or her before the disclosure of any medical information.^18^ Then the patient would be left with the option of deciding on the preferred treatment or course of action. The majority of healthcare professionals were found to be against external influence on the choice of treatment, according to studies.^17^, ^18^ They thought that only the patient and the medical team were authorized to make these. In the final decision‐making process, particularly for adult patients, parents play a passive role.^12^ This was because, if the patients were in a good enough state of mind to make the decision, they have the right to select the best course of treatment. By doing this, the patients' adherence to the treatment would be improved. In such cases of adherence, administering the chosen method would be simpler and persuading their families to respect the choice would also be easier. Close family members or the patients' parents would be consulted, if the patient is either in a vegetative state or lack the mental capacity to make decisions.^12^, ^17^, ^18^ One critical aspect is about limiting or withholding treatment.

The discussion of limiting therapy is typically brought up by medical staff. However, all involved medical and nursing staff are included in discussions about limiting therapy and keeping the families fully informed of the choices made.^24^ Typically, patients are admitted to the hospital in the hopes of recovering. Withdrawing or delaying therapy becomes the only humane course of action once it is obvious to the medical team and the family that recovery is no longer possible.^24^ Professionals take the patient's clinical condition and sociocultural context into consideration when making decisions about end‐of‐life care. Rarely do patients initiate these discussions, and professionals rarely directly inquire about the patient's preferences.^24^ Healthcare professionals perform interventions like starting artificial feeding techniques and cardiopulmonary resuscitation even in the absence of expected benefits out of fear of confrontation with family members and lawsuits.^24^

In end‐of‐life treatment, the role of medical staff includes guiding the patient to a dignified death. It is claimed that by extending the course of treatment, the patient would endure more suffering and their dignity might be compromised. The caregiver is expected to uphold this dignity, and if discontinuing treatment is the only course of action available, it must be diligently followed. The dilemma inherent in the decision‐making process for treatment withdrawal was influenced by moral values within cultural and religious perspectives. The decision would be easier if there were advanced directives/living will objecting to life‐prolonging treatment^21^, ^22^, ^25^ This could ease the decision‐making process.

Despite the right of the patient to choose the best available treatment option, euthanasia requests are infrequent. Patients do not ask for information on euthanasia because it is not yet accepted in most African cultures. There are arguments in favor of legalizing PAS/E—patients who should be given the option to decide whether they want to end their lives early, and doctors should be permitted to support them in that choice.^14^ It is recommended that a dedicated ethics team should decide which patients qualify for PAS/E.^14^, ^17^ Due to a role conflict, it should not be left up to the doctors treating the patient.^14^, ^17^

As a relationship grows between the healthcare provider as a result of medical encounters, it also grows between the provider and the patient's family members. The healthcare professional experiences the family during this time of treatment and gains empathy for what they are going through. They also patiently respond to the family's concerns and frustrations concerning the patient's condition and choices.^25^ The two key characteristics of the doctor‐patient relationship that are identified as developing through communication are empathy and trust. It takes special communication abilities to discuss end‐of‐life options and decisions with the patient. This skill set is necessary to convey confidence from the perspective of the doctors while also conveying clear information without destroying the hope held by the patient and family.

### 3.6 Decision on end of life and acceptability of euthanasia

The review found a wide variation in terms of the acceptability of PAS/E among a different set of populations.^16^ For some healthcare personnel that supported some degree of PAS/E, they are mainly concerned with the burden of a terminally ill patient on their family and the expected quality of life. This thought makes them have some sort of support for PAS.^12^, ^16^ However, there is strong opposition to PAS/E among medical doctors. The majority of them will not support PAS/E for terminally ill patients.^12^, ^16^

Euthanasia is strongly opposed by the public/lay people. Due to the sociocultural context, the procedure is typically seen as sinful or murderous.^21^, ^22^ A terminally ill person should not be helped to die, and doctors should not be permitted to help patients pass away. Because of social construction, euthanasia is viewed as murder, anyone who helps someone die is a murderer, and helping someone die is killing. There is a general belief that suffering should not warrant the right to PAS/E. Few people supported and believed in euthanasia, even though many people believed that pain was not a reason to help anyone die.^22,25^ Few people believe that euthanasia is compassionate and should be used to end the suffering of those who are in pain.^21^, ^22^, ^23^, ^25^

The idea that euthanasia should be legalized and that it can be done in certain circumstances follows support for the concept from some medical professionals.^13^ The need to alleviate patient's suffering, respect for patients' wishes (including advanced wishes), autonomy, and assisting patients in dying with dignity were all cited as reasons for this position. To prevent abuse of the procedure, safeguards or restrictions should be followed if euthanasia were to become legal. The study discovered that, despite their opposition to euthanasia, there are medical professionals that would be willing to carry it out if it were made legal.^13^

Additionally, it has been discovered that medical professionals' clinical interactions with terminally ill patients have an impact on their attitudes toward euthanasia and call for its legalization.^25^ Because they comprehend what the patients are going through, the financial and social burden the illness has placed on the patient and his family, and the suffering the patient is currently experiencing. The care providers who have spent more time with terminally ill patients tend to support PAS/E more than those who have not.^13^, ^25^

Regarding the process of terminating treatment, some healthcare professionals admit to feeling guilty. Some people experience emotional uneasiness as a result of the choice because they have been taught to preserve life and not to allow death to occur by cutting off life support. Workers in the medical field express their sorrow at the passing of a patient who had received long‐term care.^25^ The fact that the patient would die regardless of what was done for them led to despondency because it seemed like their time was wasted and that nothing could be done to prevent it. Healthcare professionals experience feelings of hopelessness based on the fact that despite all efforts to save a patient's life, treatment was discontinued because there was no chance of recovery.^25^

## 4 DISCUSSION

PAS/E involves deep ethical considerations concerning autonomy and human dignity. For instance, for the competent sick, their autonomy and dignity should be respected. This argument is in line with John Stuart Mill, Immanuel Kant, and principlism in medico‐moral ethics.^26^ The argument is that the right to self‐determination supersedes other considerations including familial consent and the duty of care principle.^27^ The overriding argument is often that autonomy (the right to self‐determination) forms the principal basis of the dignity of human nature^28^). However, experiential pain could “motivate” patients to hastily opt for PAS/E.^26^ Hence there is a need for caution in taking decisions about PAS/E despite the need to respect the “autonomy” of the sick. Beyond the ethical arguments, this review shows different positions which often characterize arguments about PAS/E.

As expected, two different positions on the acceptability of PAS were found because of the lack of consensus surrounding the issue. The prevalent view among lay people was that acceptability depends on circumstances. This result was in line with studies of a similar nature conducted in France and India, but not Kuwait. The majority view among health professionals was “never acceptable,” which is consistent with a study of a similar nature carried out among French health professionals.^29^, ^30^, ^31^, ^32^ Health professionals would run the risk of having a personal stake in the execution of PAS despite being trained to save lives rather than take them. As a result, they would be more likely than laypeople to have moral, ethical, and psychological concerns about PAS. Medical professionals' professional training has led them to oppose PAS and euthanasia when combined with sociocultural teachings.

This review clearly shows that medical students are generally opposed to euthanasia; this is not surprising. In contrast to most medical students, Radulovic and Mojsilovic^33^ observed that the majority of law and psychology students supported PAS/E. Other studies conducted among students revealed opposition to euthanasia at rates between 40% and 72%.^34^ Medical students and doctors alike are typically trained to preserve life rather than to take it. They consequently socially construct a mindset toward PAS/E. The results conformed with other studies conducted between 2006 and 2013,^35^, ^36^, ^37^ which found that most respondents opposed legalizing or engaging in PAS. The majority of British doctors in the UK oppose the legalization of PAS and euthanasia.^38^

The acceptance of euthanasia was found to be directly correlated with the professional's clinical experience, particularly with terminally ill patients, despite the strong opposition to PAS/E among medical professionals.^34^ As a result, medical students in Sudan are more willing to accept euthanasia than doctors.^15^ A study conducted in Hungary found that social science students were more accepting of assisted suicide than medical students were of the practice.^39^ Additionally, it was discovered that Mexican oncologists were much less open to euthanasia than doctors in other fields.^40^

The findings of this study about the association between age and individual acceptance of euthanasia are contradicting the findings of other studies in this area. As identified by different researchers,^41^, ^42^, ^43^ older persons are more likely than the general population to have a negative attitude regarding euthanasia. However, this study discovered a favorable association between age and euthanasia approval. This can be explained by a combination of social shame, a sense of loneliness, suffering from age‐related illnesses, and powerlessness. The socio‐cultural environment in which the elderly life plays a critical role in their attitudes toward life and decisions about the end of their lives.

The elderly require care and assistance from others because they are unable to care for themselves. Because of this dependency and continual exposure to the suffering, illness, stigmatization, identity change, and deaths of other residents, seniors may feel less in control of their lives, environments, and circumstances and more vulnerable to the pity of others. The elderly would be more exposed to suffering and the deaths of others, and because of their awareness of it and the societal context in which they live; they would be more in favor of euthanasia.

## 5 CONCLUSION

The review indicates that PAS/E involves deep ethical considerations concerning autonomy, human dignity and the preservation of life. Euthanasia continues to be controversial and debated in Africa and elsewhere. The majority of Africans hold the duty of care and preservation of life as the hallmark of medical practice. Hence, there is a wide rejection of euthanasia. Where there is some positive attitude towards euthanasia, it is strongly correlated with advanced age of the patient and the incurability of the terminal illness. However, withdrawing or withholding therapy is generally considered humane when recovery is no longer possible. Nevertheless, most health professionals do not have adequate conceptual knowledge of euthanasia. Mostly, health professionals with close contact with the terminally ill often have a positive disposition toward euthanasia, especially as a way of respecting the patient's wishes and alleviating suffering. The most observed gap is the lack of a definite care policy, medico‐legal and ethical framework in implementing withholding or withdrawal of treatment in the studies reviewed.

## AUTHOR CONTRIBUTIONS

**Jimoh Amzat**: Conceptualization; data curation; formal analysis; investigation; methodology; project administration; resources; supervision; validation; visualization; writing—original draft; writing—review and editing. **Kehinde Kazeem Kanmodi**: Conceptualization; data curation; funding acquisition; investigation; methodology; project administration; resources; software; supervision; validation; visualization; writing—original draft; writing—review and editing. **Abbas Ismail**: Data curation; formal analysis; investigation; resources. **Eyinade Adeduntan Egbedina**: Data curation; investigation; resources.

## CONFLICTS OF INTEREST STATEMENT

Kehinde Kazeem Kanmodi is an Editorial Board member of Health Science Reports and a coauthor of this article. To minimize bias, they were excluded from all editorial decision‐making related to the acceptance of this article for publication. The remaining authors declare no conflict of interest.

## ETHICS STATEMENT

Being a scoping review, ethical approval is not applicable to this study, as this study did not collect data from human or animal subjects but an open research repository.

## TRANSPARENCY STATEMENT

Kehinde Kazeem Kanmodi affirms that this manuscript is an honest, accurate, and transparent account of the study being reported; that no important aspects of the study have been omitted; and that any discrepancies from the study as planned (and, if relevant, registered) have been explained.

## Data Availability

Data sharing is not applicable to this article as no new data were created or analyzed in this study. All authors have read and approved the final version of the manuscript. Kehinde Kazeem Kanmodi had full access to all of the data in this study and takes complete responsibility for the integrity of the data and the accuracy of the data analysis.

## 1

**Table A1:** Search string for PubMed database search.

Tag	Subject search	Search String
#1	Euthanasia	((euthanasia[MeSH Terms]) OR (mercy killing[MeSH Terms])) OR (assisted suicide[MeSH Terms])
#2	African countries, dependencies, and territories	(((((((((((((((((((((((((((((((((((((((((((((((((((((((((((Algeria[MeSH Terms]) OR (Angola[MeSH Terms])) OR (Benin[MeSH Terms])) OR (Botswana[MeSH Terms])) OR (burkina faso[MeSH Terms])) OR (burundi[MeSH Terms])) OR (cabo verde[MeSH Terms])) OR (cape verde[MeSH Terms])) OR (cameroon[MeSH Terms])) OR (central african republic[MeSH Terms])) OR (chad[MeSH Terms])) OR (comoros[MeSH Terms])) OR (congo[MeSH Terms])) OR (ivory coast[MeSH Terms])) OR (cote d ivoire[MeSH Terms])) OR (djibouti[MeSH Terms])) OR (democratic republic of congo[MeSH Terms])) OR (egypt[MeSH Terms])) OR (equatorial guinea[MeSH Terms])) OR (eritrea[MeSH Terms])) OR (eswatini[MeSH Terms])) OR (ethiopia[MeSH Terms])) OR (gabon[MeSH Terms])) OR (gambia[MeSH Terms])) OR (ghana[MeSH Terms])) OR (guinea[MeSH Terms])) OR (guinea bissau[MeSH Terms])) OR (kenya[MeSH Terms])) OR (lesotho[MeSH Terms])) OR (liberia[MeSH Terms])) OR (libya[MeSH Terms])) OR (madagascar[MeSH Terms])) OR (malawi[MeSH Terms])) OR (mali[MeSH Terms])) OR (mauritania[MeSH Terms])) OR (mauritius[MeSH Terms])) OR (morocco[MeSH Terms])) OR (mozambique[MeSH Terms])) OR (namibia[MeSH Terms])) OR (niger[MeSH Terms])) OR (nigeria[MeSH Terms])) OR (rwanda[MeSH Terms])) OR (sao tome and principe[MeSH Terms])) OR (senegal[MeSH Terms])) OR (seychelles[MeSH Terms])) OR (sierra leone[MeSH Terms])) OR (somalia[MeSH Terms])) OR (south africa[MeSH Terms])) OR (south sudan[MeSH Terms])) OR (sudan[MeSH Terms])) OR (tanzania[MeSH Terms])) OR (togo[MeSH Terms])) OR (tunisia[MeSH Terms])) OR (uganda[MeSH Terms])) OR (zambia[MeSH Terms])) OR (zimbabwe[MeSH Terms])) OR (reunion[MeSH Terms])) OR (saint helena[MeSH Terms])) OR (western sahara[MeSH Terms])) OR (mayotte[MeSH Terms])
#3	#1 AND #2	(#1) AND (#2)

**Table A2:** Search string for SCOPUS database search.

Tag	Subject search	Search string
#1	Euthanasia	(TITLE‐ABS‐KEY (euthanasia) OR TITLE‐ABS‐KEY (mercy AND killing) OR TITLE‐ABS‐KEY (assisted AND suicide))
#2	African countries, dependencies, and territories	((TITLE‐ABS‐KEY (angola) OR TITLE‐ABS‐KEY (benin) OR TITLE‐ABS‐KEY (botswana) OR TITLE‐ABS‐KEY (burkina AND faso) OR TITLE‐ABS‐KEY (burundi) OR TITLE‐ABS‐KEY (cameroon) OR TITLE‐ABS‐KEY (cabo AND verde) OR TITLE‐ABS‐KEY (cape AND verde) OR TITLE‐ABS‐KEY (central AND african AND republic) OR TITLE‐ABS‐KEY (chad) OR TITLE‐ABS‐KEY (comoros) OR TITLE‐ABS‐KEY (congo) OR TITLE‐ABS‐KEY (ivory AND coast) OR TITLE‐ABS‐KEY (democratic AND republic AND of AND congo) OR TITLE‐ABS‐KEY (djibouti) OR TITLE‐ABS‐KEY (equatorial AND guinea) OR TITLE‐ABS‐KEY (eritrea) OR TITLE‐ABS‐KEY (ethiopia) OR TITLE‐ABS‐KEY (gabon) OR TITLE‐ABS‐KEY (gambia) OR TITLE‐ABS‐KEY (ghana) OR TITLE‐ABS‐KEY (guinea) OR TITLE‐ABS‐KEY (guinea‐bissau) OR TITLE‐ABS‐KEY (kenya) OR TITLE‐ABS‐KEY (lesotho) OR TITLE‐ABS‐KEY (liberia) OR TITLE‐ABS‐KEY (madagascar) OR TITLE‐ABS‐KEY (malawi) OR TITLE‐ABS‐KEY (mali) OR TITLE‐ABS‐KEY (mauritania) OR TITLE‐ABS‐KEY (mauritius) OR TITLE‐ABS‐KEY (mayotte) OR TITLE‐ABS‐KEY (mozambique) OR TITLE‐ABS‐KEY (namibia) OR TITLE‐ABS‐KEY (niger) OR TITLE‐ABS‐KEY (nigeria) OR TITLE‐ABS‐KEY (reunion) OR TITLE‐ABS‐KEY (rwanda) OR TITLE‐ABS‐KEY (saint AND helena) OR TITLE‐ABS‐KEY (sao AND tome AND principe) OR TITLE‐ABS‐KEY (senegal) OR TITLE‐ABS‐KEY (seychelles) OR TITLE‐ABS‐KEY (sierra AND leone) OR TITLE‐ABS‐KEY (somalia) OR TITLE‐ABS‐KEY (south AND africa) OR TITLE‐ABS‐KEY (south AND sudan))) OR ((TITLE‐ABS‐KEY (eswatini) OR TITLE‐ABS‐KEY (togo) OR TITLE‐ABS‐KEY (uganda) OR TITLE‐ABS‐KEY (zambia) OR TITLE‐ABS‐KEY (zimbabwe) OR TITLE‐ABS‐KEY (egypt) OR TITLE‐ABS‐KEY (libya) OR TITLE‐ABS‐KEY (algeria) OR TITLE‐ABS‐KEY (tunisia) OR TITLE‐ABS‐KEY (morocco) OR TITLE‐ABS‐KEY (western AND sahara) OR TITLE‐ABS‐KEY (sudan) OR TITLE‐ABS‐KEY (tunisia)))
#3	#1 AND #2	(#1) AND (#2)

**Table A3:** Search string for other database (AMED—The allied and complementary medicine database; APA psycInfo and CINAHL complete) search via EBSCO interface.

Tag	Subject search	Search string
#1	Euthanasia	AB Euthanasia OR AB Mercy killing OR AB Assisted suicide
#2	African countries, dependencies, and territories	AB algeria OR AB angola OR AB benin OR AB botswana OR AB burkina faso OR AB burundi OR AB cape verde OR AB cabo verde OR AB cameroon OR AB central african republic OR AB chad OR AB comoros OR AB congo OR AB cote d'ivoire OR AB ivory coast OR AB djibouti OR AB democratic republic of congo OR AB egypt OR AB equatorial guinea OR AB eritrea OR AB eswatini OR AB ethiopia OR AB gabon OR AB gambia OR AB ghana OR AB guinea OR AB guinea bissau OR AB kenya OR AB lesotho OR AB liberia OR AB libya OR AB madagascar OR AB malawi OR AB mali OR AB mauritania OR AB mauritius OR AB morocco OR AB mozambique OR AB namibia OR AB niger OR AB nigeria OR AB rwanda OR AB (sao tome and principe) OR AB senegal OR AB seychelles OR AB sierra leone OR AB somalia OR AB south Africa OR AB south sudan OR AB sudan OR AB tanzania OR AB togo OR AB tunisia OR AB uganda OR AB zambia OR AB zimbabwe OR AB reunion OR AB saint helena OR AB western sahara OR AB mayotte
#3	#1 AND #2	#1 AND #2

**Table A4:** List of literature considered for full‐text screening and screening outcome.

S/N	Literature considered for full text screening	Outcomes concerning inclusion into the review	
	.	Included	Excluded (with reasons)
1	Kpanake L, Dassa KS, Sorum PC, Mullet E. Togolese lay people's and health professionals' views about the acceptability of physician‐assisted suicide. J Med Ethics. 2014 Sep;40(9):621‐4. doi: 10.1136/medethics‐2013‐101424. Epub 2013 Jul 31. PMID: 23903992.	Yes	
2	Ahmed AM, Kheir MM. Attitudes towards euthanasia among final‐year Khartoum University medical students. East Mediterr Health J. 2006 May‐Jul;12(3‐4):391‐7. PMID: 17037708.	Yes	
3	Ahmed AM, Kheir MM, Abdel Rahman A, Ahmed NH, Abdalla ME. Attitudes towards euthanasia and assisted suicide among Sudanese doctors. East Mediterr Health J. 2001 May;7(3):551‐5. PMID: 12690779.	Yes	
4	Hosking M, Whiting G, Brathwate C, Fox P, Boshoff A, Robbins L. Cultural attitudes towards death and dying: a South African perspective. Palliat Med. 2000 Sep;14(5):437‐9. doi: 10.1191/026921600701536147. PMID: 11064796.		Yes (wrong publication type)
5	Turner JS, Michell WL, Morgan CJ, Benatar SR. Limitation of life support: frequency and practice in a London and a Cape Town intensive care unit. Intensive Care Med. 1996;22(10):1020‐5. doi: 10.1007/BF01699222. PMID: 8923064.	Yes	
6	Wainer S, Khuzwayo H. Attitudes of mothers, doctors, and nurses toward neonatal intensive care in a developing society. Pediatrics. 1993;91(6):1171‐5. PMID: 8502523.	Yes	
7	Meyers DH. Views of SA doctors regarding active medical help in dying. Med J Aust. 1993 Apr 19;158(8):577. doi: 10.5694/j.1326‐5377.1993. tb121893.x. PMID: 8397517.		Yes (Wrong publication type)
8	Luna‐Meza A, Godoy‐Casasbuenas N, Calvache JA, Díaz‐Amado E, Gempeler Rueda FE, Morales O, Leal F, Gómez‐Restrepo C, de Vries E. Decision making in the end‐of‐life care of patients who are terminally ill with cancer ‐ a qualitative descriptive study with a phenomenological approach from the experience of healthcare workers. BMC Palliat Care. 2021;20(1):76. doi: 10.1186/s12904‐021‐00768‐5. PMID: 34049535; PMCID: PMC8164310.		Yes (wrong study population)
9	Kpanake L, Pineda Marín C, Pajot E, Muñoz Sastre MT, Mullet E. Functional measurement in the Field of Empirical Ethics. Avances en Psicología Latinoamericana. 2021 Dec;39(3).		Yes (published in non‐English language)
10	Pinto E, Marcos G, Walters C, Gonc¸alves F, Sacarlal J, Castro L, et al. (2020) Palliative care in Mozambique: Physicians' knowledge, attitudes and practices. PLoS ONE 15(8): e0238023. https://doi.org/10.1371/journal.pone.0238023	Yes	
11	Jacobs RK, Hendricks M. Medical students' perspectives on euthanasia and physician‐assisted suicide and their views on legalizing these practices in South Africa. S Afr Med J. 2018 May 25;108(6):484‐489. doi: 10.7196/SAMJ.2018.v108i6.13089. PMID: 30004328.	Yes	
12	Burger H, Gwyther L, Krause R, Ratshikana‐Moloko M, Hellenberg D. Medical students' perceptions on euthanasia and physician‐assisted suicide: Are they fully informed? S Afr Med J. 2018 Aug 30;108(9):12424. doi: 10.7196/SAMJ.2018.v108i9.13528. PMID: 30182889.		Yes (wrong publication type)
13	Daeschler M, Verdino RJ, Caplan AL, Kirkpatrick JN. Defibrillator Deactivation against a Patient's Wishes: Perspectives of Electrophysiology Practitioners. Pacing Clin Electrophysiol. 2015 Aug;38(8):917‐24. doi: 10.1111/pace.12614. Epub 2015 Mar 16. PMID: 25683098.		Yes (wrong outcomes)
14	Olasunkanmi A. Euthanasia and the Experiences of the Yoruba People of Nigeria. Ethics & Medicine. 2015;31(1):31‐38.		Yes (wrong publication type)
15	Kalanzi DJ. The controversy over euthanasia in Uganda: a case of the Baganda. International Journal of Sociology and Social Policy. 2013; 33(3/4):203‐217. doi: 10.1108/01443331311308249	Yes	
16	Tadros G, Rakhawy MY, Khoweiled A, El‐Houssini AM, Khan F. Perception of physician‐assisted suicide among Egyptian psychiatrists: cultural perspective. The Psychiatrist. 2011;35(1):15‐8. doi:10.1192/pb. bp.110.030411	Yes	
17	Lerato MA, Maku MM, Makwela SA, Seoka PG, Prinsloo EA, Bachmann OM, Joubert G. Palliative care‐‐attitudes and practices of Bloemfontein general practitioners and medical officers. S Afr Med J. 2006;96(7):569‐70; discussion 570. PMID: 16909169.		Yes (wrong outcome)
18	van Rooyen D, Elfick M, Strümpher J. Registered nurses' experience of the withdrawal of treatment from the critically ill patient in an intensive care unit. Curationis. 2005 Feb;28(1):42‐51. doi: 10.4102/curationis.v28i1.920. PMID: 15850152.	Yes	
19	Nortje N, de Vos HM, le Roux MC. The attitudes of older South Africans towards euthanasia. Social Work/Maatskaplike Werk. 2004;40(3). doi: 10.15270/40‐3‐333.	Yes	
20	Gharoro ET, Igberase GO, Okubor PO, Onakewhor JU. Caring for the terminally ill: what do the doctors think? Afr J Med Med Sci. 2003;32(4):377‐380.	Yes	
21	Van Der Geest S. ‘I want to go!’ How older people in Ghana look forward to death. Ageing & Society. 2002;22(1):7‐28. doi:10.1017/S0144686X02008541.		Yes (wrong study design)
22	Adamolekun, K. (1998). Openness of Health Professionals about Death and Terminal Illness in a Nigerian Teaching Hospital. OMEGA ‐ Journal of Death and Dying, 36(1), 23–32. https://doi.org/10.2190/5F95-L2F6-ELR6-0466		Yes (wrong outcomes)
23	Brits L, Human L, Pieterse L, Sonnekus P, Joubert G. Opinions of private medical practitioners in Bloemfontein, South Africa, regarding euthanasia of terminally ill patients. J Med Ethics. 2009 Mar;35(3):180‐2. doi: 10.1136/jme.2008.027417. PMID: 19251970.	Yes	
24	Vandewiele M. Attitudes of Senegalese secondary school students toward death. OMEGA J Death Dying. 1984;14(4):329‐34.		Yes (wrong outcomes)
25	Guana EW, Louw J, Manganyi NC. Thoughts about death and dying in an African sample. OMEGA J Death Dying. 1990;20(3):245‐58. doi: 10.2190/PRXN‐6YHE‐D36U‐G7T0	Yes	
